# Evolution of treatment practices and outcomes in multiple myeloma during 2013–2022: a Finnish real world registry study

**DOI:** 10.2340/1651-226X.2025.42647

**Published:** 2025-05-05

**Authors:** Anu Partanen, Marika Waltari, Johanna Vikkula, Riikka Mattila, Katja Närhi, Jonna Eeva, Mervi Putkonen

**Affiliations:** a Department of Medicine, Kuopio University Hospital, Kuopio, Finland; b Johnson & Johnson, Espoo, Finland; c Medaffcon Oy, Espoo, Finland; d Department of Medicine, Tampere University Hospital, Tampere, Finland; e Hematology and Stem Cell Transplant Unit, Turku University Hospital, Turku, Finland

**Keywords:** real-world, multiple myeloma, outcome, survival, treatment, novel therapy

## Abstract

**Background and purpose:**

Multiple myeloma (MM) is a heterogenous hematologic malignancy with an evolving treatment landscape. This Finnish real-world evidence study clarifies the evolution of treatment practices and outcomes over recent years.

**Methods:**

This retrospective analysis included 1,733 patients with MM diagnosed between 2013 and 2022. The cohort was identified and electronic health record data were collected from four hospital data lakes and linked to national registries, covering 54% of Finland’s population. Patients were divided by stem cell transplantation (SCT) status into a SCT group (512 patients) and a non-SCT group (1,221 patients), and further by diagnosis period (2013–2017 vs. 2018–2022).

**Results:**

The average age of the patients was 71.3 years at diagnosis. Novel therapeutic use markedly increased during the follow-up, especially lenalidomide as part of frontline and maintenance therapy in SCT patients. For SCT patients the 4-year survival rate improved from 81.7% (95% confidence interval [CI]: 76.4–86.0) in 2013–2017 to 93.0% (95% CI: 87.0–96.3) in 2018–2022. For non-SCT-patients, the median overall survival (OS) increased slightly from 41.3 months (95% CI: 38.1–45.6) in the 2013–2017 period to 43.8 months (95% CI: 39.8–55.3) in the 2018–2022 period, although the difference was not statistically significant. High risk cytogenetics and high International Staging System class appeared to persist as factors indicating shorter OS.

**Interpretation:**

While advancements of novel drugs have resulted in a notable survival benefit for patients undergoing SCT, the survival of non-SCT-patients has remained comparatively static. New approaches in the treatment of MM for elderly and frail non-SCT patients are warranted.

## Introduction

Multiple myeloma (MM) is a B-cell neoplasms characterized by overproduction of monoclonal immunoglobulins, or their light chains found in blood and/or urine. In Finland, MM is the second most prevalent hematologic cancer, with 383 new diagnoses reported in 2021 [1]. In the 21st century a significant evolution in MM treatments has been seen due to introduction of new drug classes such as proteasome inhibitors (bortezomib, carfilzomib, ixazomib), immunomodulatory drugs (thalidomide, lenalidomide, pomalidomide), and anti-CD38 monoclonal antibodies (daratumumab, isatuximab) [2].These agents have been integrated into triplet or quadruplet combinations followed by autologous stem cell transplant (ASCT) in transplant eligible patients. The treatment landscape continues to advance rapidly because several new treatment modalities such as chimeric antigen receptor T-cell therapy (CAR-T) and bispecific antibodies are available.

Along with these treatment advances, the treatment patterns and prevailing practices have become complex and also highly heterogenous [3–6]. This complexity escalates with each subsequent line of therapy. For younger patients eligible for stem cell transplant (SCT) international guidelines recommend induction therapy consisting of triplet or quadruplet combinations of bortezomib, lenalidomide, thalidomide, anti-CD38 monoclonal antibodies, cyclophosphamide and dexamethasone, followed by SCT, consolidation and maintenance therapy [4–6]. For individuals ineligible for SCT, a first-line treatment consisting of duplet or triplet combinations of anti-CD38 monoclonal antibodies, bortezomib, lenalidomide, melphalan and dexamethasone are suggested. An alternative regimen for older, frail patients includes melphalan with prednisone or cyclophosphamide with prednisone [7]. If the disease progresses, treatment selection is guided by specific disease and patient-related factors.

Despite the recent advances in relapsed or refractory MM treatment, the prognosis for patients remains poor. The depth and duration of response to current therapies decrease over time and the disease inevitably becomes refractory to the treatment options [7, 8]. Moreover, MM is a heterogenous disease with variable clinical presentation, cytogenetic findings as well as responses to treatment. It is therefore essential to follow the real-world practices closely.

A series of recent real-world evidence (RWE) studies internationally and in Finland have investigated treatment and outcomes of real world MM patients [9–19]. However, the swift pace of advancements in MM therapy underscores the necessity for more current investigations to capture the dynamic nature of the disease’s management.

This non-interventional, retrospective RWE study investigated the largest Finnish MM cohort to date with the aim to describe the changing treatment landscape, and outcomes for patients in four of the five University Hospital districts in Finland, linked with data from national registries.

## Materials and methods

This was a retrospective registry-based study at Helsinki and Uusimaa (HUS; population base approximately 1.5 million), Hospital District of Southwest Finland (HDSF; 0.5 million), Pirkanmaa Hospital District (PHD; 0.5 million) and Hospital District of North Savon (HDNS; 0.25 million) between the years 2013 and 2022. Areas were selected based on data availability. All patients treated for MM in these areas are included in respective data lakes. For the identified cohort, clinical data, such as diagnoses, laboratory values, genetics and procedure codes was collected and linked to electronic health records and other hospital databases via the respective data lakes. Data on primary care diagnoses and procedures was collected from the Care register for healthcare (AvoHILMO), reimbursed drug purchases from the Social Insurance Institution (SII), dates of death from Statistics Finland, and an age and sex matched control cohort from Digital and population services agency (DVV).

### Cohort formation

The study cohort included all adult patients diagnosed with MM (ICD-10: C90) in specialized care in 2013–2022, and who received treatment for myeloma, and who lived in one of the four hospital districts at the end of follow-up. Patients with AL-amyloidosis (ICD-10: E85) were excluded. From HDNS, only patients diagnosed in 2015–2022 were included due to data availability reasons. For each MM patient, two age, sex and site-matched controls were identified for comparison of overall survival (OS). MM patients were followed from the diagnosis of MM until death, or end of study (EOS; December 31, 2022), whichever came first. The index dates of the controls were set to the same date as the index of corresponding case (date of MM diagnosis). MM patients were stratified by SCT status (SCT and non-SCT) based on performed procedures during the follow-up, and by diagnosis year (2013–2017 and 2018–2022), whereas those groups were not directly compared. See Supplementary Methods for more details.

### Treatment lines

The treatment lines of MM were constructed according to the current international guidelines [20]. Firstly, all records within 90 days from each other were considered to belong to same treatment line. If the same treatment regimen was started again after a pause, these were merged into one continuous treatment line regardless of the length of the pause. Secondly, if a new drug was added to the treatment after 2 months, this was considered as unplanned addition and thus, a start of a new treatment line. Finally, unusual treatment regimens were inspected and adjusted manually. See Supplementary Methods for more details.

For patients undergoing SCT, the initial phase of treatment comprises induction therapy, transplantation, and possible consolidation and maintenance therapies. In this study this sequence was combined into one course of treatment called ‘SCT-treatment’. Subsequent therapies were analyzed as treatment lines according to guidelines. In this fashion, the first treatment after the ‘SCT-treatment’ roughly corresponds to treatment of first relapse.

### Statistical analyses

Demographical (age, sex) and clinical (immunoglobulin type, cytogenetic changes, International Staging System; ISS, and co-diagnoses) variables were summarized at MM diagnosis as mean with standard deviation (SD) for the continuous variables and number and proportion of patients for the categorical variables. The difference between strata was tested using chi-squared/Fisher’s exact test (categorical variables) and *t*-test (continuous variables).

Treatment outcomes were assessed using time to event analysis, namely Kaplan-Meier fit (OS) and competing risk model (time to next treatment; TTNT). OS was defined as time from MM diagnosis to death (event) or EOS (censoring event). For patients with SCT, OS was also assessed from first SCT to death (event) or EOS (censoring event) to remove the immortal survival bias from MM diagnosis until the transplantation. TTNT was defined as time from initiation of a treatment until initiation of the next treatment (event), death (competing risk), or EOS (censoring event). Median TTNT were reported from the event-free survival curves, which represent a composite endpoint combining the initiation of the next treatment and death as competing risks. Difference between strata was tested using log-rank test. In addition, the association of baseline characteristics with OS among patients with SCT was assessed using a Cox proportional hazards model. Cox model was not performed for the non-SCT patients as the model assumptions were violated.

All analyses were performed with R version 4.0.3.

## Results

We identified 1,733 patients diagnosed and having received treatment for MM during 2013–2022. Of these, 512 patients had records of undergoing SCT, with either autologous or allogeneic SCT. The remaining 1,221 patients received treatment without SCT (non-SCT) (Table 1).

**Table 1:** Characteristics of MM patients ± 3 months from index.

		Overall	SCT				non-SCT				Missing %

		2013–2022	2013–2022	2013–2017	2018–2022	*P* *	2013–2022	2013–2017	2018–2022	*P* *	
***N***		1,733	512	252	260		1221	577	644		
**Age, years, mean (SD)**		69.9 (11.0)	60.5 (8.4)	60.0 (7.5)	60.9 (9.1)	0.200	73.9 (9.4)	73.4 (9.3)	74.3 (9.4)	0.120	0
**Age group <60**		313 (18.1)	218 (42.6)	113 (44.8)	105 (40.4)	**< 0. 001**	95 (7.8)	41 (7.1)	54 (8.4)	**0.033**	
**61–69**		476 (27.5)	252 (49.2)	132 (52.4)	120 (46.2)		224 (18.3)	115 (19.9)	109 (16.9)		
**70–79**		653 (37.7)	42 (8.2)	7 (2.8)	35 (13.5)		611 (50.0)	303 (52.5)	308 (47.8)		
**≥ 80**		291 (16.8)	0 (0.0)	0 (0.0)	0 (0.0)		291 (23.8)	118 (20.5)	173 (26.9)		
**Sex, female, *N* (%)**		834 (48.1)	241 (47.1)	128 (50.8)	113 (43.5)	0.116	593 (48.6)	279 (48.4)	314 (48.8)	0.933	0
**MM type**	**IgG**	731 (53.8)	240 (55.2)	120 (57.4)	120 (53.1)	0.813	491 (53.1)	203 (49.3)	288 (56.1)	0.080	21.5
	**IgA**	292 (21.5)	88 (20.2)	41 (19.6)	47 (20.8)		204 (22.1)	92 (22.3)	112 (21.8)		
	**IgD**	21 (1.5)	10 (2.3)	< 5	< 10		11 (1.2)	< 10	< 5		
	**IgM**	16 (1.2)	0 (0.0)	0 (0.0)	0 (0.0)		16 (1.7)	9 (2.2)	7 (1.4)		
	**light chain**	300 (22.1)	97 (22.3)	44 (21.1)	53 (23.5)		203 (21.9)	100 (24.3)	103 (20.1)		
**ISS**	**I**	224 (16.5)	118 (25.4)	66 (29.1)	52 (21.8)	0.153	106 (11.9)	43 (10.9)	63 (12.7)	0.618	21.8
	**II**	602 (44.4)	212 (45.6)	95 (41.9)	117 (49.2)		390 (43.8)	178 (45.3)	212 (42.6)		
	**III**	530 (39.1)	135 (29.0)	66 (29.1)	69 (29.0)		395 (44.3)	172 (43.8)	223 (44.8)		
**High risk cytogenetic changes, *N* (%)#**		224 (22.8)	95 (25.5)	40 (23.3)	55 (27.5)	0.414	129 (21.1)	57 (22.0)	72 (20.4)	0.702	43.2
**Length of the follow-up, months, mean (SD)**		39.4 (29.9)	53.5 (29.1)	73.7 (26.5)	34.0 (14.7)	**< 0.001**	33.4 (28.2)	46.3 (32.5)	21.9 (16.7)	**< 0.001**	0

* *P*-value for difference between patients diagnosed between 2013–2017 and 2018–2022. Values in bold indicate statistically significant (p<0.05).

# Cytogenetic changes are missing from HDNS.

The median age at diagnosis was 71.3 (interquartile range [IQR]: 63.3, 77.6). Patients who underwent SCT were notably younger, with a median age of 61.7 (IQR: 55.7, 66.9), compared to 74.9 (IQR: 69.7, 79.8) for those in the non-SCT group. Among the SCT recipients, 498 had records of ASCT, and 42 of allogeneic SCT (AlloSCT). Patients receiving AlloSCT were younger, with a median age of 50.5 years (IQR: 45.2, 55.1), compared to those receiving ASCT who had a median age of 62.8 years (IQR: 56.8, 67.3). For characteristics of the SCT group stratified by type of SCT, please see Supplementary Table 1. The most prevalent monoclonal protein type was IgG, observed in 53.8% of patients. Notably, the ISS and the revised ISS (R-ISS) scores were elevated in patients who did not undergo SCT. In addition, several comorbidities and codiagnoses were more frequent in the non-SCT group (Supplementary Table 2). Overall, no major differences were seen in patient characteristics between early and late cohorts as presented in Table 1 or between study areas, as presented in Supplementary Table 3. More comprehensive patient characteristics are presented in Supplementary Table 4.

**Figure 1:**
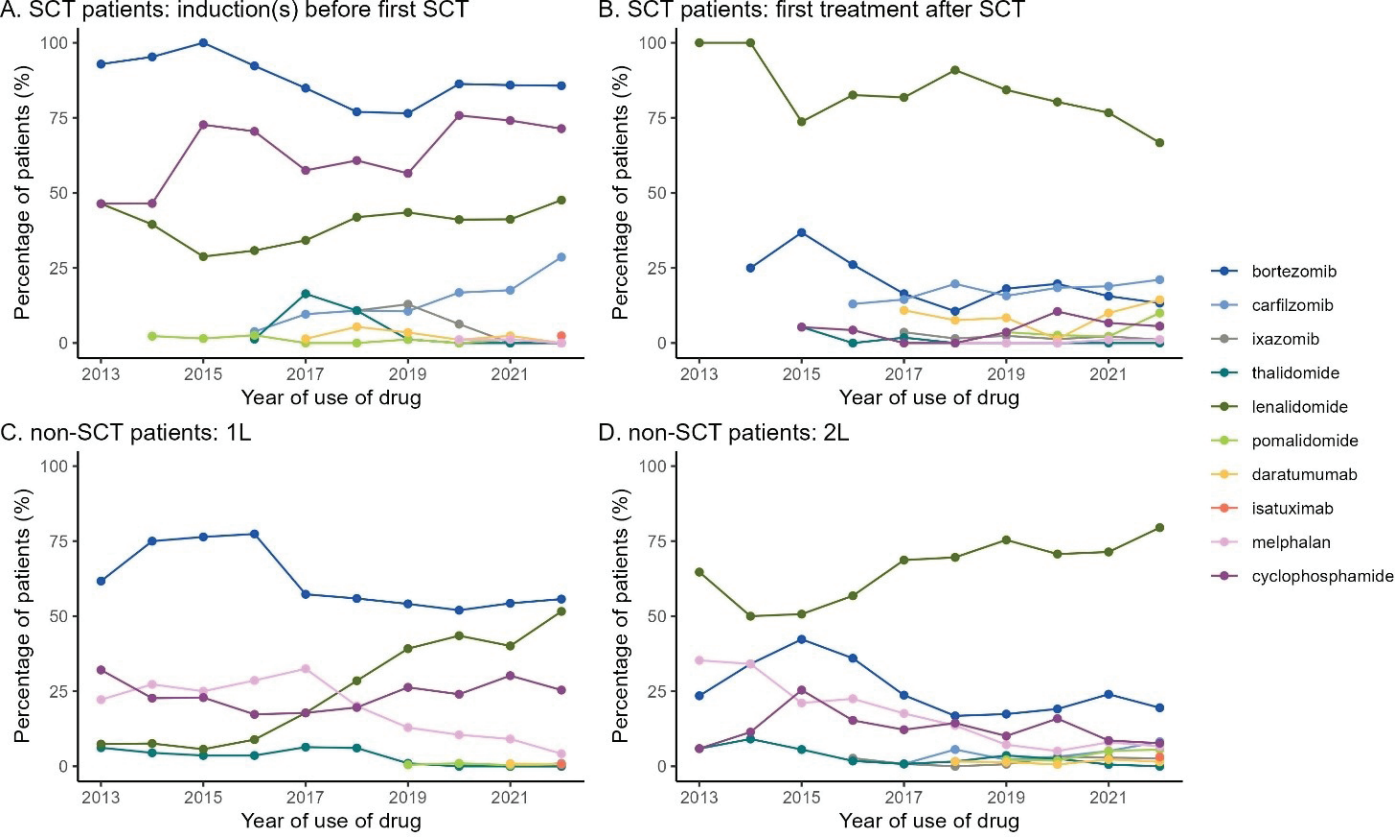
Trends in the use of MM drugs for SCT patients (upper panels) and non-SCT patients (lower panel). For SCT patients, the left panel represents induction treatment(s) and the right panel represents the first treatment line after SCT treatment. Note that consolidation and maintenance therapies are not depicted here but are presented in Supplementary Figure 1. For non-SCT patients, the left panel shows 1L (first-line) treatment and the right panel shows 2L (second-line) treatment. MM: Multiple myeloma; SCT: stem cell transplantation.

Trends in the use of MM drugs are depicted by yearly usage pattern in Figure 1 and by diagnosis year in Supplementary Table 5, regardless of their combination. Notably, the treatment paradigm has undergone significant shifts. For SCT patients bortezomib has remained a standard induction therapy, though its use in later lines has markedly decreased. This decrease has been accompanied by the increased use of more novel drugs such as lenalidomide and karfilzomib in induction and anti-CD38 monoclonal antibodies as well as pomalidomide in relapsed setting. The use of ixazomib and thalidomide is waning. Consolidation and maintenance therapies were adopted during the study period, as visualized in Supplementary Figure 1. In recent years, most patients have received maintenance therapy after SCT.

For non-SCT patients, the changes have been more modest. These patients are treated more conservatively, with wide use of bortezomib and especially lenalidomide based treatment across treatment lines. The use of melphalan has decreased, while the use of pomalidomide, carfilzomib and ixazomib has increased in the later lines. However, the use of anti-CD38 based treatment remains scarce.

**Figure 2:**
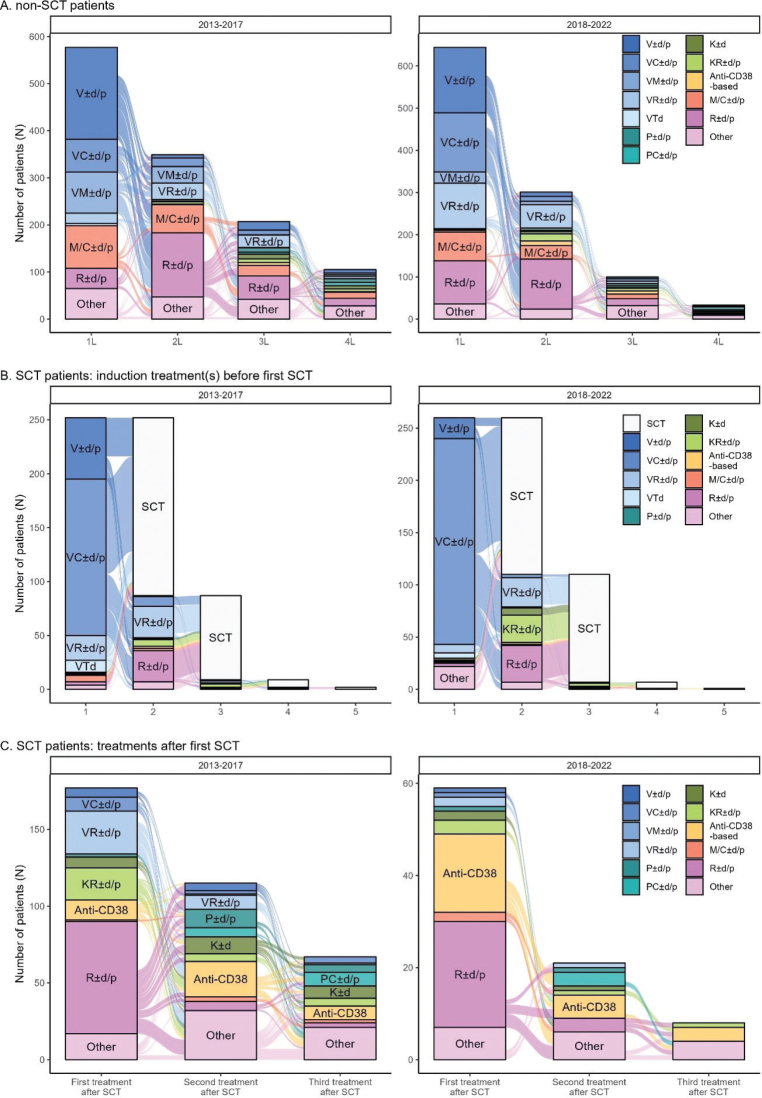
Sankey plots of four first treatment lines for non-SCT patients (A), SCT-patients before the first SCT (B), and SCT-patients after the first SCT (C). SCT: stem cell transplantation.

MM treatment was then studied per combination (Figure 2) stratified by year of diagnosis. The number of distinct treatment regimens used by at least four patients was 45. Non-SCT patients are mostly treated with doublet therapies (one active MM substance combined with dexamethasone or prednisone). While the use of lenalidomide combinations has increased in earlier treatment lines, only modest other changes were observed between time windows.

Induction treatments prior to SCT (Figure 2B) and treatment lines after the first SCT-treatment (Figure 2C) were analyzed separately to visualize the complex treatment patterns of SCT patients in the frontline. SCT patients are mostly treated with bortezomib-based triplet combinations. The same trend of increased use and earlier positioning of novel therapies is seen particularly in the post-SCT setting, with lenalidomide and anti-CD38 based combinations taking a place in early treatment after SCT. Notably, 35% of SCT patients diagnosed during 2013–2017 received two or more induction treatments before SCT, which increased to 42% for patients diagnosed during 2018–2022. The use of carfilzomib in the second induction can be seen for the latter cohort.

No major changes in treatment patterns were observed between study areas (Supplementary Figures 2–4). Treatment patterns with detailed treatment combinations are shown in Supplementary Figure 5.

Analysis of the TTNT (Supplementary Figure 6) showed no significant change between the two time periods for non-SCT patients in the first treatment line (log rank *p* = 0.65). However, for SCT patients, TTNT (i.e. to first treatment after the initial SCT) increased significantly from 37.3 months (95% confidence interval [CI]: 32.8, 44.7) to not reached (log rank *p* < 0.001). At 48 months, 42.2% (95% CI: 36.4, 48.8) of the patients diagnosed during 2013–2017 and 63.2% (95% CI: 55.1, 72.5) of patients diagnosed during 2018–2022 were alive and were not given treatment after SCT.

**Figure 3:**
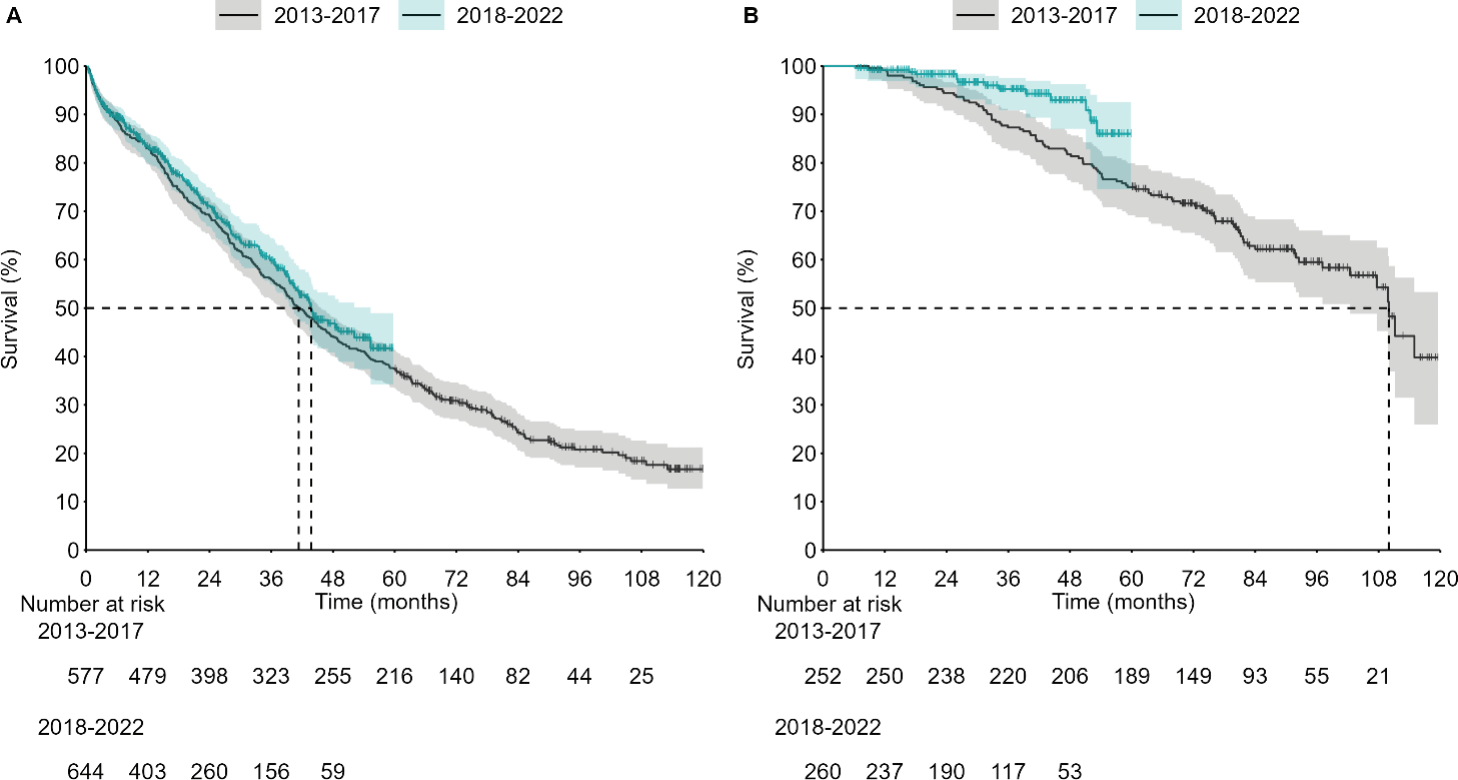
OS of non-SCT (A) and SCT (B) patients during 2013–2017 (black lines) and 2018–2022 (blue lines). Shaded areas represent 95% CI. OS: overall survival; SCT: stem cell transplantation; CI: confidence interval.

Next, we analyzed the OS of SCT and non-SCT patients, stratified by the year of diagnosis. For non-SCT patients, the median OS was 41.3 months (95% CI: 38.1–45.6) for those diagnosed between 2013 and 2017 (Figure 3A). This slightly increased to 43.8 months (95% CI: 39.8–55.3) for patients diagnosed from 2018 onwards, although the difference was not statistically significant. In contrast, SCT patients showed a notable improvement in survival. The 4-year survival rate for patients diagnosed between 2013 and 2017 was 81.7% (95% CI: 76.4–86.0) and the rate improved to 93.0% (95% CI: 87.0–96.3) for those diagnosed between 2018 and 2022, as depicted in Figure 3B.

Using SCT as a stratifying factor introduces an immortal survival bias from index date to SCT. If this immortal time differs between time periods, it may bias the results. The median time from index to SCT has remained stable between the two time periods (190 days (IQR: 157–261) for patients diagnosed during 2013–2017 and 198 days (IQR: 164–243) for patients diagnosed during 2018–2022). When OS was analyzed from the timepoint of SCT onwards, a similar increase in OS was observed (Supplementary Figure 7). OS was similar for both ASCT and allo-SCT patients (log rank *p* = 0.205). No major differences were observed between hospital districts in OS for either cohort (Supplementary Figure 8).

**Figure 4:**
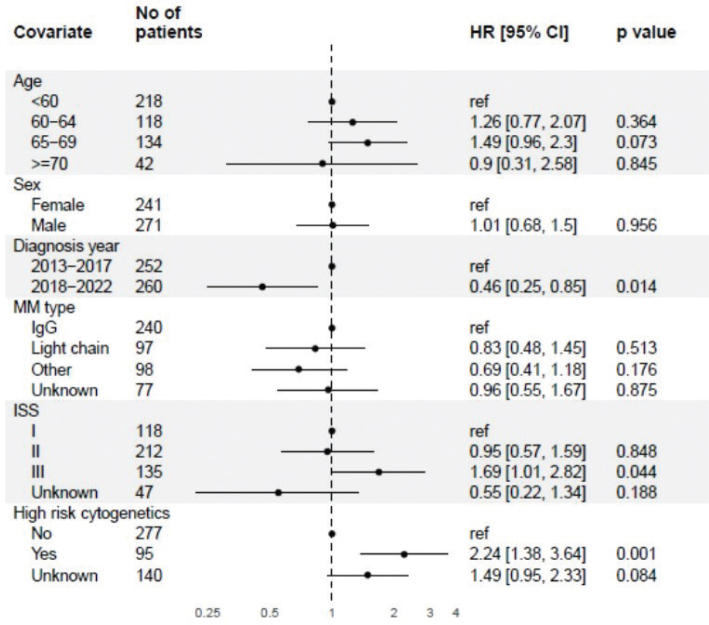
Cox proportional hazards model of OS for SCT-receiving patients. OS: overall survival; SCT: stem cell transplantation.

A Cox proportional hazards model for OS and adjusting for age, sex, diagnosis year, MM type, ISS and high risk cytogenetics revealed that patients with ISS-class III and those with high-risk cytogenetics were associated with an increased risk of mortality (Figure 4). Conversely, a diagnosis made between 2018 and 2022, as opposed to between 2013 and 2017, was significantly associated with a reduced risk of death.

**Figure 5:**
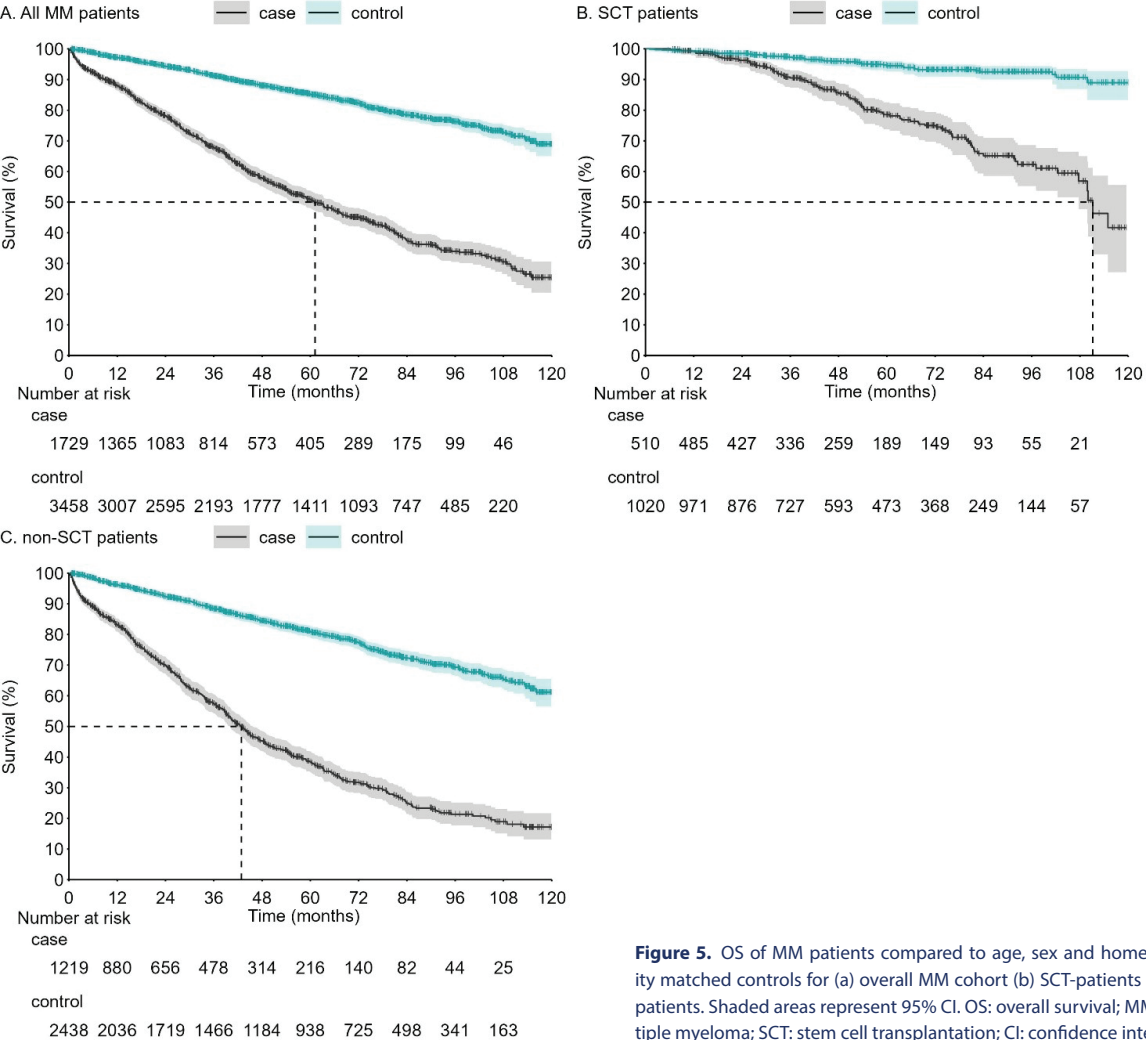
OS of MM patients compared to age, sex and home municipality matched controls for (a) overall MM cohort (b) SCT-patients (c) non-SCT patients. Shaded areas represent 95% CI. OS: overall survival; MM: MM: Multiple myeloma; SCT: stem cell transplantation; CI: confidence interval.

To contextualize the survival outcomes of MM patients against the general Finnish population, we conducted a matched comparison. Each MM patient was paired with two Finnish individuals sharing the same sex, age, and home municipality. The comparative survival analysis is depicted in Figure 5. The 4-year survival rate for the control group was 88.0% (95% CI: 86.7–89.2), while it was only 57.9% (95% CI: 55.1–60.5) for MM patients. When the analysis was stratified by SCT status, the 4-year survival for SCT patients was 85.7% (95% CI: 81.8–88.8) compared to 95.9% (95% CI: 94.3–97.1) for matched controls. For non-SCT patients, the 4-year survival was 45.3% (95% CI: 42.0–48.6) compared to 84.4% (95% CI: 82.6–86.0) for matched controls.

## Discussion

The aim of this retrospective study, which included 1,733 patients, was to evaluate how the introduction and earlier use of novel therapies have impacted survival and treatment patterns for both SCT and non-SCT patients over recent years.

The patient- and region-based differences in treatment choices resulted in complex treatment pathways. Treatment decisions are often influenced by reimbursement policies and previous treatments received, as per both Finnish and international guidelines [4, 6]. This complexity is further increased by Finland’s dual-channel system for medication reimbursement, where different reimbursement authorities approve pharmacy dispensed medications (typically oral) vs. treatments provided in hospital.

In both SCT and non-SCT groups bortezomib and lenalidomide have predominated in the treatment of myeloma across both the early (2013–2017) and late (2018–2022) cohorts. There has been an increase in the use of multiple induction therapies before SCT, aiming for deeper treatment responses before transplantation. After the first relapse, there is a trend toward using more novel therapies, such as anti-CD38 therapies, as noted also by Ruotsalainen et al. [12], Loponen et al. [11] and Vikkula et al. [9]. Despite this, lenalidomide-based treatments remain the most common. Pomalidomide has also been added to later treatment lines according to reimbursement policies.

Notably, some treatments, such as IRd (ixazomib with lenalidomide and dexamethasone) for SCT-patients during 2018–2022, and VRd (bortezomib with lenalidomide and dexamethasone) may include patients involved in related trials before reimbursement of those combinations [21, 22]. Overall, the treatment of MM is moving toward a more personalized approach, where patient profile, risk factors, intolerances, previous treatments as well as patient preference and reimbursement decisions play a crucial role, especially in the later treatment lines.

Despite the advanced age of most MM patients, the disparity in 4-year OS between MM patients (57.9%), and controls (88.0%) underscores that their life expectancy is significantly reduced. However, SCT patients diagnosed between 2018 and 2022 showed a marked improvement in 4-year survival from 81.7% to 93.0%. In agreement, SCT patients also show a marked increase in TTNT for their SCT treatment from 37.3 months to not reached. This improvement is likely linked to the utilization of novel drugs, as well as lenalidomide maintenance therapy after SCT and is in line with Canadian findings [23]. While the non-SCT cohort showed a marginal increase in median OS over time, the lack of statistical significance suggests that advancements in treatment have not dramatically altered survival for this group. This highlights the need for continued innovation and adaptation of new therapeutic strategies for non-SCT patients.

Recent studies from Finland have suggested OS ranges from 4.1 years to 6.1 years (13–15), compared to our 5.1 years. However, variations in study approaches and follow-up times (up to 7 years) make comparisons inconclusive. No significant differences were found between hospital districts in our study.

The integration of Finnish hospital data lakes with comprehensive clinical data and national registries, alongside universally accessible healthcare constitutes a robust framework for conducting real-world studies. There are some limitations in the study: the possibility of incomplete data may have led to a small subset of patients being misclassified as not having received SCT when they actually have. Moreover, some patients diagnosed with MM close to EOS may have received SCT after the EOS, thus appearing incorrectly as non-SCT patients in the study data. Similarly, patients eligible to SCT who die before receiving SCT automatically end up in the non-SCT cohort. However, this is anticipated to have a minimal impact on the study’s outcomes. The definition of treatment lines post hoc from registry data are difficult and may not be entirely consistent with treatment lines considered by the treating clinicians. Despite this challenge, we believe that the figures and tables presented here accurately represent the treatment landscape of MM during the study period in Finland. The cohort from HDNS was available only from 2015, causing a small bias with the lack of early patients from this region. However, with HDNS contributing a minority of patients for the study, this effect is likely small. For cohorts with fewer than five patients, specific patient counts are withheld in compliance with data privacy laws; consequently, certain results are presented as censored. The findings of this study are likely applicable to nations with comparable demographics, healthcare frameworks, and therapeutic protocols.

In conclusion, this comprehensive retrospective analysis of MM patients highlights the significant impact of novel drugs and evolving treatment paradigms on outcome principally in SCT patients. As the field of MM continues to advance, new approaches in the treatment of MM are warranted also for the elderly and frail non-SCT patients to bridge the survival gap and enhance quality of life.

## Supplementary Material

Evolution of treatment practices and outcomes in multiple myeloma during 2013–2022: a Finnish real world registry study

Evolution of treatment practices and outcomes in multiple myeloma during 2013–2022: a Finnish real world registry study

Evolution of treatment practices and outcomes in multiple myeloma during 2013–2022: a Finnish real world registry study

## Data Availability

This study is based on secondary use of healthcare register data. Hence, data cannot be shared openly.
