# Supplementary material for: Evolution of treatment practices and outcomes in multiple myeloma during 2013–2022: a Finnish real world registry study
Source: Acta Oncol. 2025 May 5;64:42647. doi: 10.2340/1651-226X.2025.42647 (PMC12067984; doi:10.2340/1651-226X.2025.42647)
Supplement: Evolution of treatment practices and outcomes in multiple myeloma during 2013–2022: a Finnish real world registry study [file AO-64-42647-s1.pdf]

Supplementary Table 1. Characteristics for SCT patients stratified by auto vs. allo-SCT.

Variable	Estimate/level	Overall	AlloSCT	ASCT	p	Missing %
**n**		512	42	470		
**Age at index, years, median (IQR)**		62 [56, 67]	51 [45, 55]	63 [57, 67]	<0.001	0
**Sex, N (%), female, N (%)**		241 (47)	19 (45)	222 (47)	0.931	0
**MM type, N (%)**	IgA or IgD	98 (23)	7 (18)	91 (23)	0.553	15
	IgG	240 (55)	20 (53)	220 (55)		
	light chain	97 (22)	11 (29)	86 (22)		
**ISS, N (%)**	I	118 (25)	12 (30)	106 (25)	0.734	9
	II	212 (46)	18 (45)	194 (46)		
	III	135 (29)	10 (25)	125 (29)		
**RISS, N (%)**	I	56 (14)	<5	52-55	0.507	20
	II	290 (71)	29 (81)	261 (70)		
	III	64 (16)	<5	60-63		
**High risk cytogenetic changes, N (%)**		95 (26)	11 (32)	84 (25)	0.453	27
**Length of follow-up, months, median (IQR)**		49 [30, 76]	74 [38, 93]	48 [28, 74]	0.006	0

Supplementary Table 5. Trends in usage of MM drugs in SCT and non-SCT patients diagnosed during 2013-2017 and 2018-2022.

	Treatment sequence/line	Total N per LOT		bortezomib		carfilzomib[#]		ixazomib[#]		thalidomide		lenalidomide		pomalidomide[#]		daratumumab[#]		isatuximab[#]	
		2013-2017	2018-2022	2013-2017	2018-2022	2013-2017	2018-2022	2013-2017	2018-2022	2013-2017	2018-2022	2013-2017	2018-2022	2013-2017	2018-2022	2013-2017	2018-2022	2013-2017	2018-2022
SCT, N (%)	SCT[&]	252	260	240 (95.2)*	234 (90.0)*	19 (7.5)*	64 (24.6)*	<5*	23 (8.8)*	32 (12.7)*	7 (2.7)*	126 (50.0)*	234 (90.0)*	<5	<5	<5	5 (1.9)	0 (0.0)	<5
	1st after SCT[&]	177	59	51 (28.8)*	5 (8.5)*	35 (19.8)	17 (28.8)	5 (2.8)*	7 (11.9)*	<5	0 (0.0)	138 (78.0)*	34 (57.6)*	<5*	9 (15.3)*	13 (7.3)*	14 (23.7)*	0 (0.0)*	5 (8.5)*
	2nd after SCT[&]	115	21	28 (24.3)	<5	23 (20.0)	6 (28.6)	19 (16.5)	<5	<5	0 (0.0)	44 (38.3)	7 (33.3)	35 (30.4)*	12 (57.1)*	22 (19.1)	6 (28.6)	<5	<5
	3rd after SCT[&]	67	8	5 (7.5)	0 (0.0)	20 (29.9)	<5	11 (16.4)	<5	0 (0.0)	<5	22 (32.8)	<5	25 (37.3)	<5	8 (11.9)	<5	<5*	<5*
non-SCT, N (%)	1	577	644	386 (66.9)	438 (68.0)	0 (0.0)	<5	0 (0.0)	<5	23 (4.0)*	<5*	66 (11.4)*	219 (34)*	0 (0.0)	<5	0 (0.0)	<5	0 (0.0)	<5
	2	349	301	104 (29.8)	90 (29.9)	7 (2.0)*	27 (9.0)*	7 (2.0)	12 (4.0)	12 (3.4)	<5	202 (57.9)*	206 (68.4)*	<5*	18 (6)*	<5*	7 (2.3)*	0 (0.0)*	6 (2.0)*
	3	207	100	59 (28.5)	19 (19)	20 (9.7)	12 (12.0)	15 (7.2)*	16 (16.0)*	8 (3.9)	<5	112 (54.1)	46 (46.0)	19 (9.2)*	24 (24.0)*	<5*	7 (7.0)*	<5	<5
	4	105	33	21 (20.0)	6 (18.2)	17 (16.2)	7 (21.2)	8 (7.2)*	7 (21.2)*	0 (0.0)	0 (0.0)	39 (37.1)	6 (18.2)	23 (21.9)*	19 (57.6)*	<5	0 (0.0)	0 (0.0)*	<5*

**Chi-squared/Fisher's exact test p<0.05 for comparison of different time frames per drug and per treatment number/line. [#]novel therapies, [&]For SCT patients, treatments are sequenced as described in methods, i.e. "SCT" comprises induction(s), SCT, and possible consolidation and maintenance therapies, "1st after SCT" indicates the first therapy after the "SCT" phase (i.e. roughly corresponds to treatment to first relapse), and so on.*
