# Supplementary material for: Evolution of treatment practices and outcomes in multiple myeloma during 2013–2022: a Finnish real world registry study
Source: Acta Oncol. 2025 May 5;64:42647. doi: 10.2340/1651-226X.2025.42647 (PMC12067984; doi:10.2340/1651-226X.2025.42647)
Supplement: Evolution of treatment practices and outcomes in multiple myeloma during 2013–2022: a Finnish real world registry study [file AO-64-42647-s3.pdf]

Supplementary material has been published as submitted. It has not been copyedited, or typeset by Acta Oncologica

Supplementary information

Supplementary table 1. (separate Excel)

Supplementary table 2. (separate Excel)

Supplementary table 3. (separate Excel)

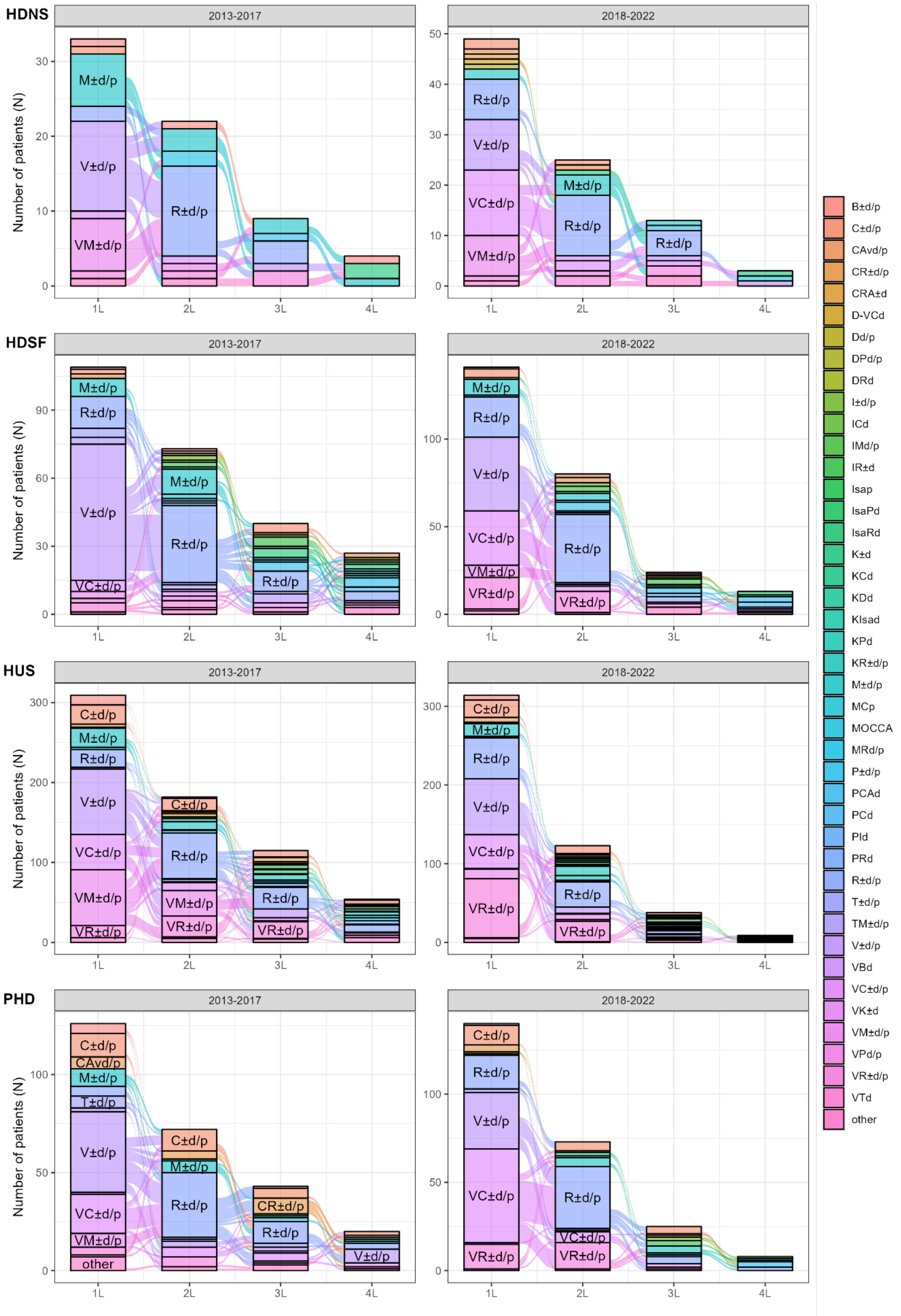

Supplementary Figure 1. *Sankey plots of non-SCT patients divided by year of treatment initiation and hospital district.*

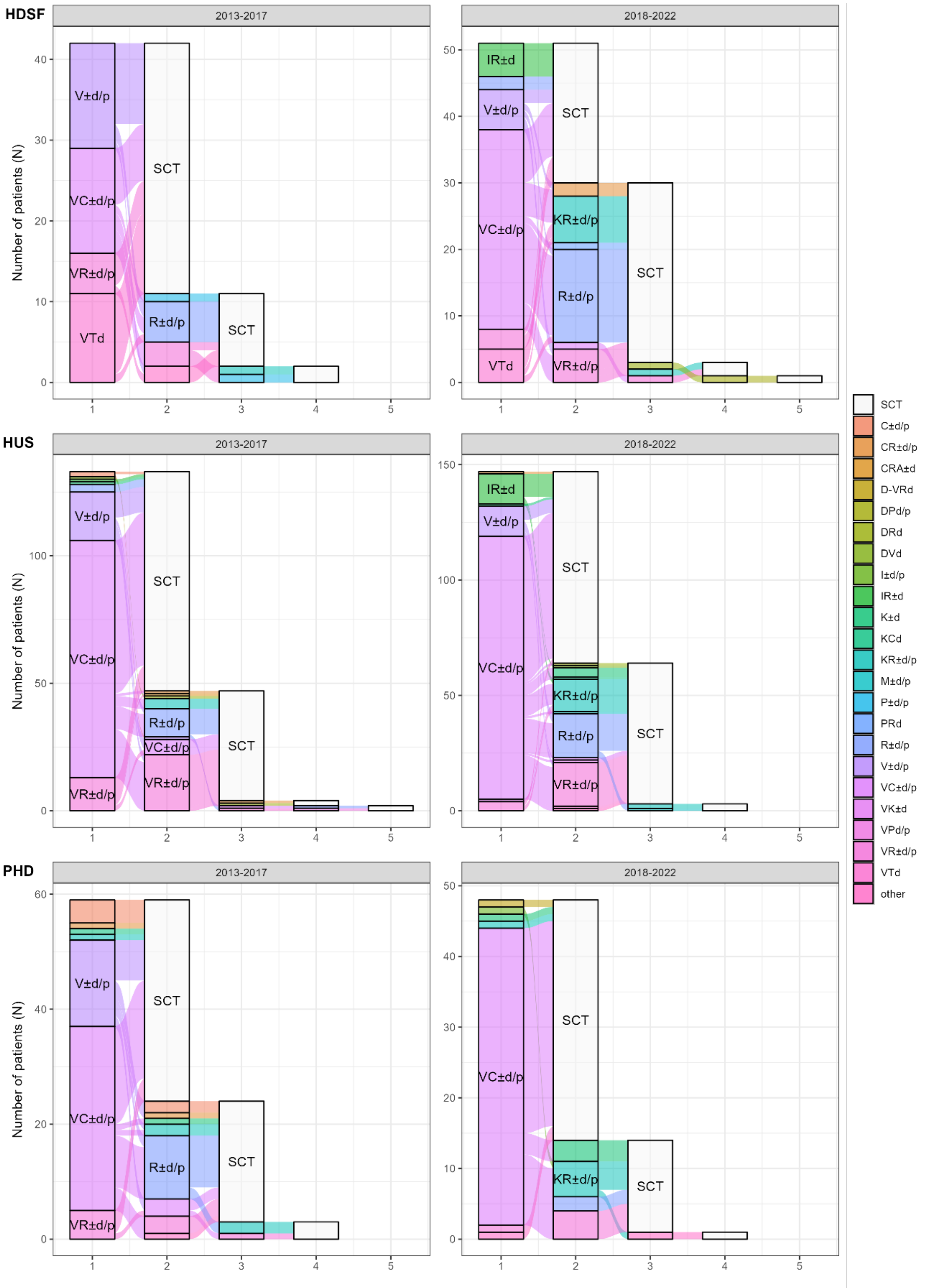

Supplementary Figure 2. *Sankey plots of SCT- patients induction treatment divided by year of treatment initiation and hospital district. Due to privacy regulations and small patient numbers patients from HDNS cannot be shown.*

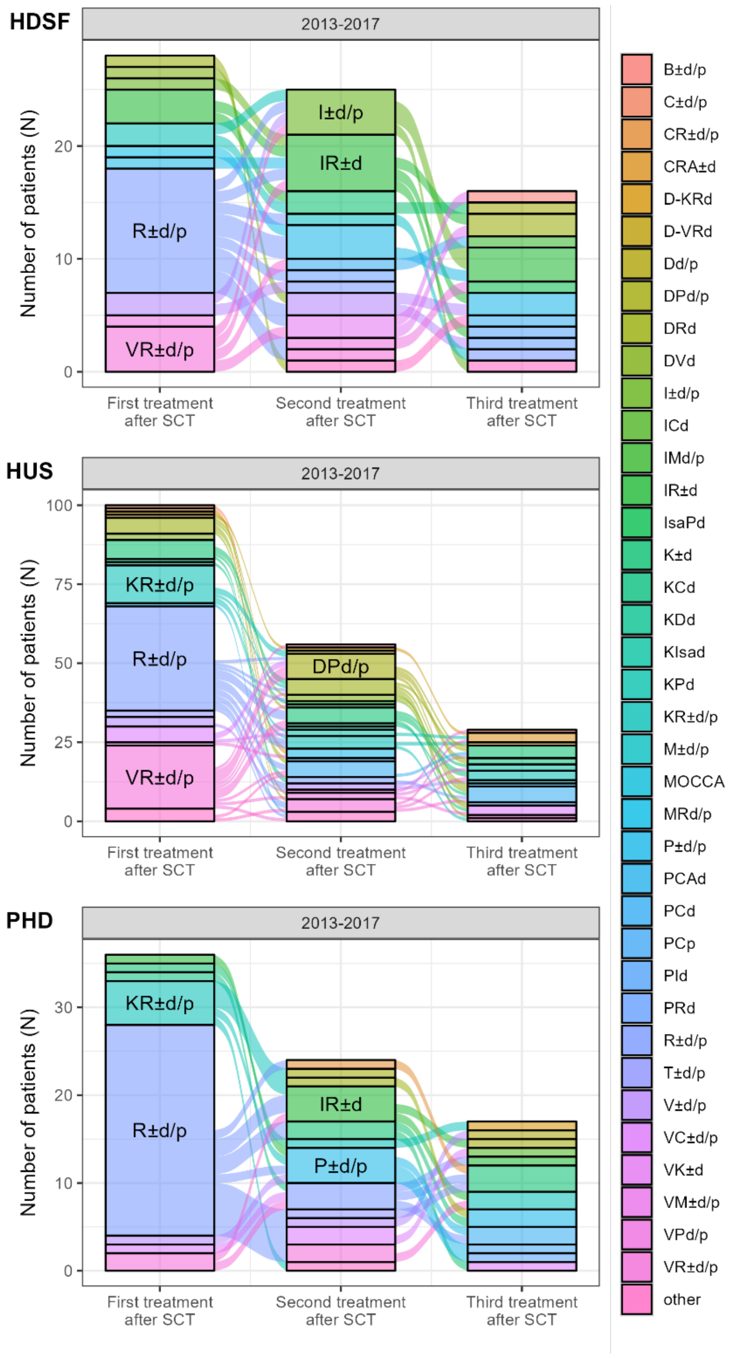

Supplementary Figure 3. *Sankey plots of treatment of SCT-patients after SCT during 2013-2017 per hospital district. Due to privacy regulations and small patient numbers patients during 2018-2022 and from HDNS cannot be shown.*

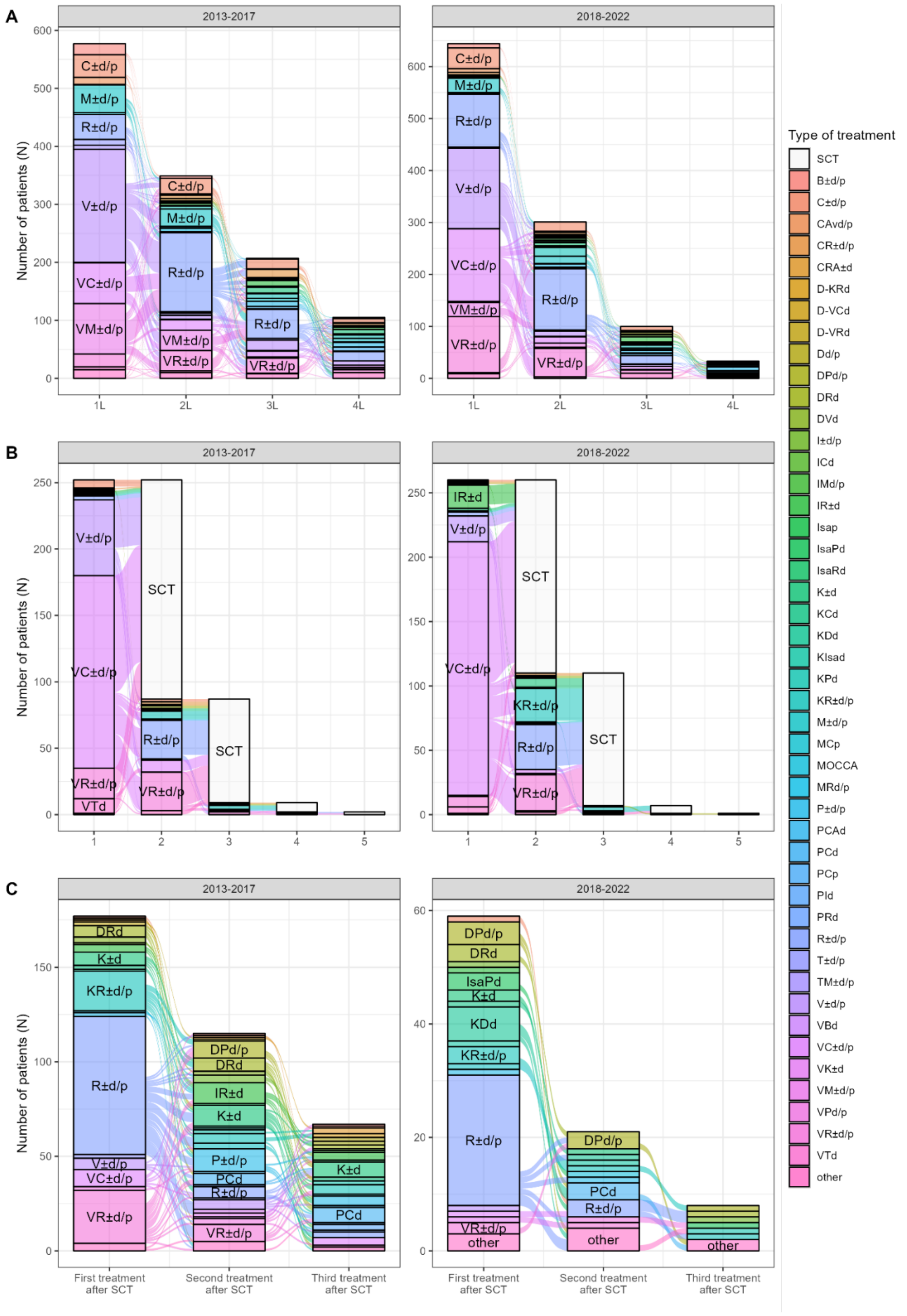

Supplementary Figure 4*. Sankey plots of treatment lines for non-SCT patients (A), SCT-patients before the first SCT (B), and SCT-patients after the first SCT (C) with detailed description of various treatment combinations.*

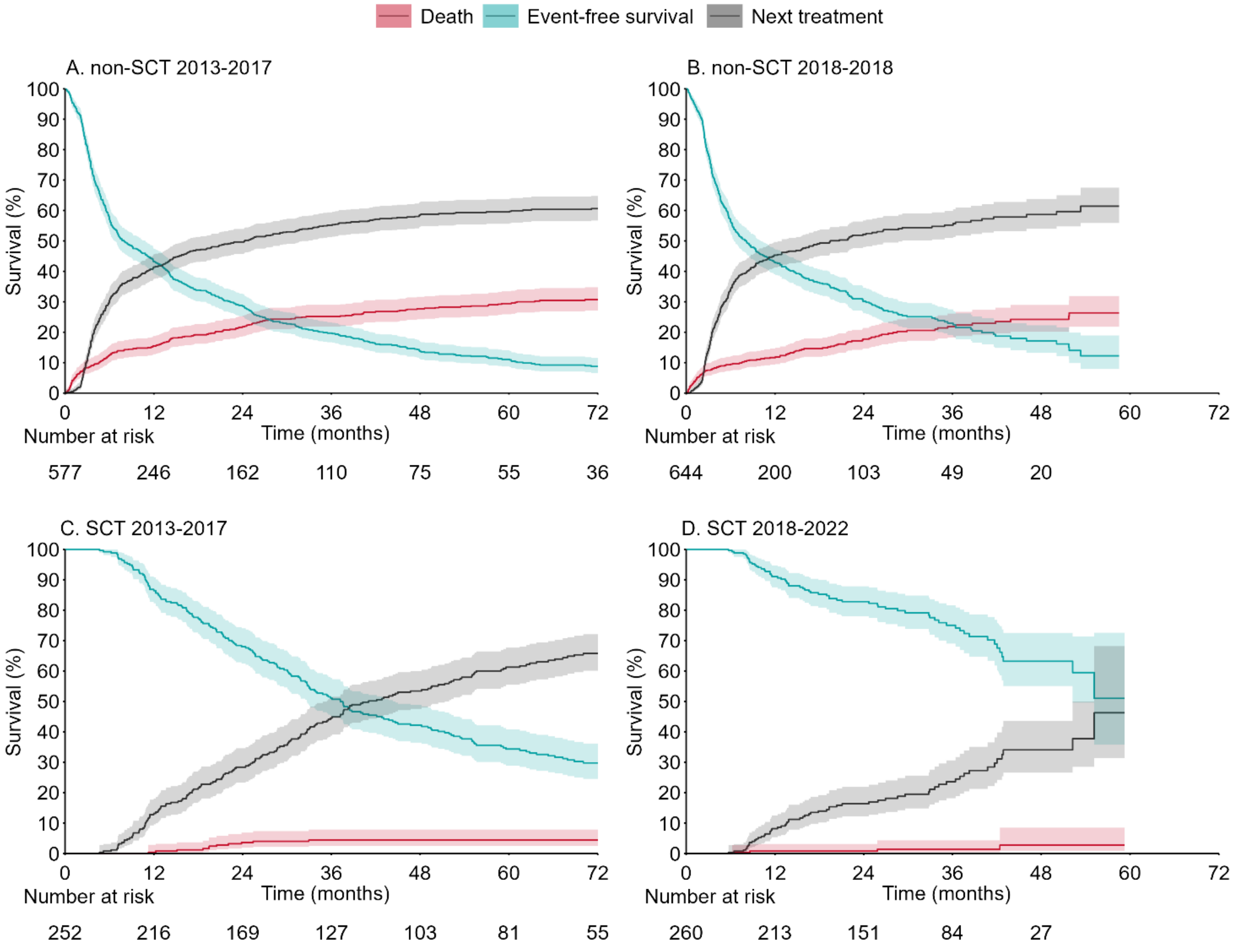

Supplementary Figure 5. *Competing risk of beginning of next treatment (black line) or death (red line), whichever comes first. The blue line denotes patients who have neither commenced further treatment nor succumbed to the disease. For non-SCT patients (Panels A and B), the graph displays the time to the second line of treatment. For SCT patients (Panels C and D), it shows the time to the first line of treatment after the initial SCT. Panels A and C correspond to patients diagnosed between 2013-2017, while Panels B and D relate to those diagnosed between 2018-2022. Shaded areas represent 95% CI.*

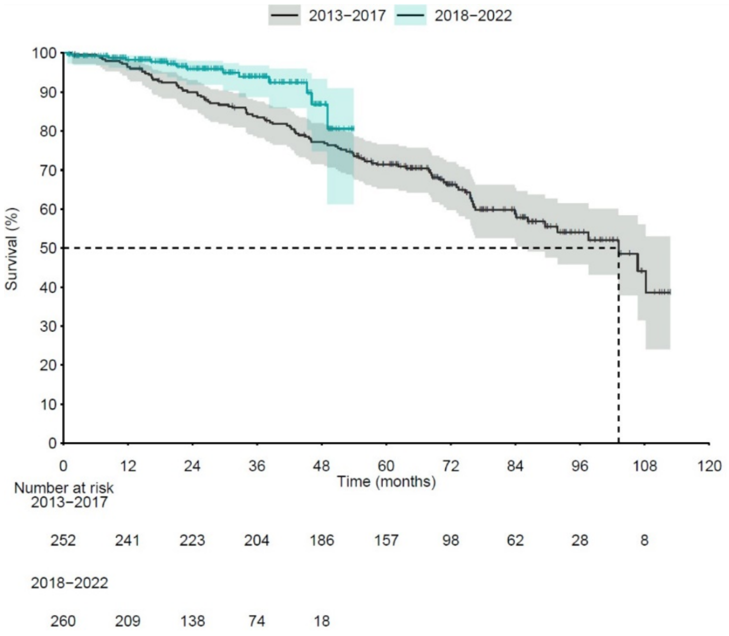

Supplementary figure 6. *OS for SCT patients from date of receiving SCT to death. Black line: patients diagnosed between 2013-2017, blue line: patients diagnosed between 2018-2022. Shaded areas represent 95% CI.*

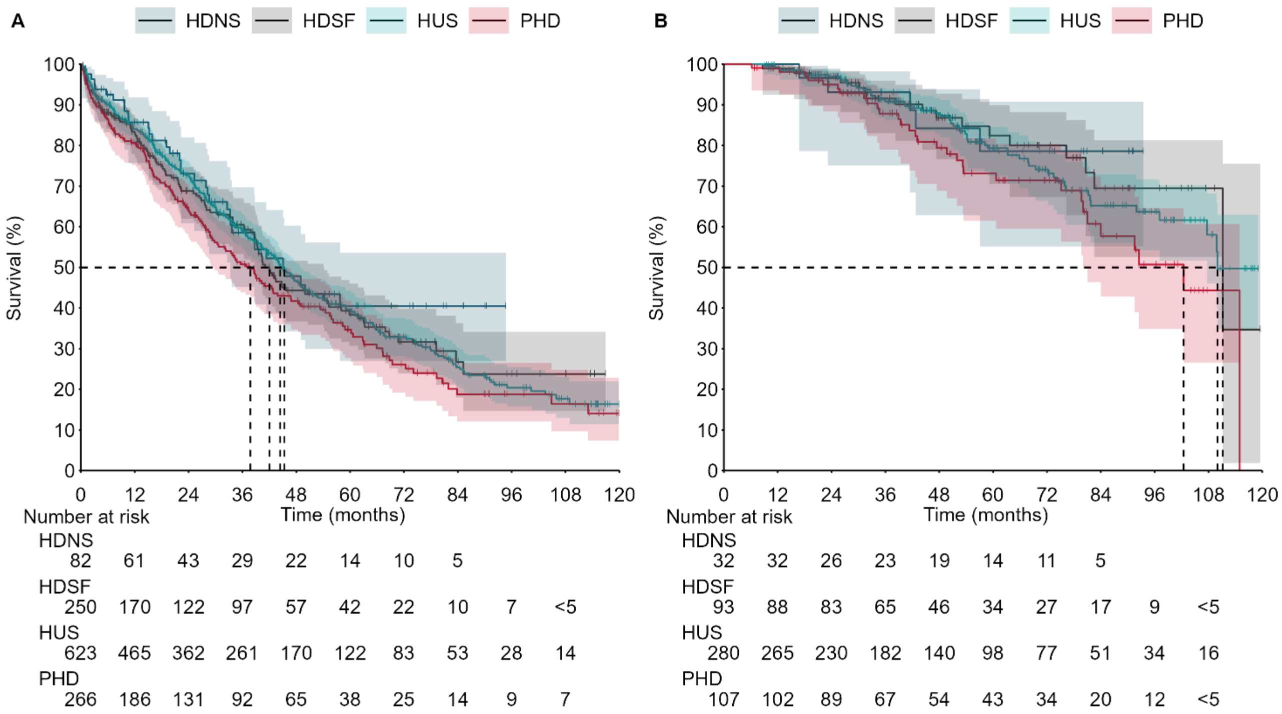

Supplementary Figure 7. *OS for non-SCT (A) and SCT (B) patients by hospital district. Shaded areas represent 95% CI.*

Supplemental methods

Stratification: Patients with SCT were identified based on performed procedures (procedure codes: WW300, WW302, WW304, and WW306) and records of previous transplants (ICD-10: Z94.8 with description of type and date of SCT) during the follow-up. Note that a few patients diagnosed with MM close to EOS may have received SCT after EOS, and thus seem in the study data incorrectly as non-SCT patients. When stratified by diagnosis year, patients diagnosed in 2013-2017 were followed until death or EOS, i.e. they were not censored at the end of 2017. Thus, for example, some of the presented treatments may have started 2018 or later.

Characteristics: For clinical variables, data max ±3 months from diagnosis was used. If multiple records were available per patient within that time window, the record closest to index was utilized. Co-diagnoses were reported from the baseline (5 years prior to index) and from the follow-up complemented with baseline data (data from 5 years prior to index until EOF). All diagnoses recorded at primary and specialized care were used.

Treatment lines: For patients with SCT, consolidation therapy (VC±d/p, VT±d/p, KR±d/p) was required to start within 4 months from the transplant and maintenance therapy (V, R, K, VR, KR) within 2 months from the end of the consolidation. If consolidation was not given, maintenance therapy was required to start within 4 months from the transplant. The start and end dates of the treatments/treatment lines were defined based on the records of the MM specific drugs, i.e. the records of the supportive medications (dexamethasone and prednisone) were not used to identify the start and end dates of the regimens.

To visualize treatment patterns, similar treatment combinations with or without dexamethasone or prediso(lo)ne were grouped to one treatment option and marked as ±d/p. Also, all treatment combinations with daratumumab and isatuximab were grouped as “Anti-CD38-based” therapy, and melphalane and cyclophosphamide therapies were combined as M/C±d/p. Most common treatment combinations were visualized as is and the rest were grouped as Other.
